# Epstein-Barr virus, interleukin-10 and multiple sclerosis: A ménage à trois

**DOI:** 10.3389/fimmu.2022.1028972

**Published:** 2022-10-07

**Authors:** Günther Schönrich, Mohammed O. Abdelaziz, Martin J. Raftery

**Affiliations:** ^1^ Institute of Virology, Charité– Universitätsmedizin Berlin, Freie Universität Berlin, Humboldt-Universität zu Berlin, Berlin Institute of Health, Berlin, Germany; ^2^ Department of Hematology, Oncology and Tumor Immunology (CCM), Charité– Universitätsmedizin Berlin, Freie Universität Berlin, Humboldt-Universität zu Berlin, Berlin Institute of Health, Berlin, Germany

**Keywords:** viral IL-10, IL-10, antiviral immune responses, viral immune evasion, virus-induced immunopathogenesis, multiple sclerosis, viruses

## Abstract

Multiple Sclerosis (MS) is an autoimmune disease that is characterized by inflammation and demyelination of nerve cells. There is strong evidence that Epstein-Barr virus (EBV), a human herpesvirus infecting B cells, greatly increases the risk of subsequent MS. Intriguingly, EBV not only induces human interleukin-10 but also encodes a homologue of this molecule, which is a key anti-inflammatory cytokine of the immune system. Although EBV-encoded IL-10 (ebvIL-10) has a high amino acid identity with its cellular counterpart (cIL-10), it shows more restricted and partially weaker functionality. We propose that both EBV-induced cIL-10 and ebvIL-10 act in a temporally and functionally coordinated manner helping the pathogen to establish latency in B cells and, at the same time, to balance the function of antiviral T cells. As a result, the EBV load persisting in the immune system is kept at a constant but individually different level (set point). During this immunological tug of war between virus and host, however, MS can be induced as collateral damage if the set point is too high. Here, we discuss a possible role of ebvIL-10 and EBV-induced cIL-10 in EBV-driven pathogenesis of MS.

## Introduction

Epstein–Barr virus (EBV), a human gammaherpesvirus, persists in 95% of the world population due to sophisticated immune evasion strategies (1). After transmission of EBV to naïve individuals, the first site of replication is in the orophayryngeal epithelium (2). Subsequently, the virus crosses the mucosal barrier, infects naïve B cells, and establishes latency within a few days post infection (3). Latency requires expression of viral proteins that induce proliferation, growth transformation and differentiation of naïve B cells into memory B cells (4, 5). EBV persists lifelong as episomal DNA in resting memory B cells that percolate through lymphoid tissue and are detectable in the circulation (6). After stimulation, latently infected memory B cells terminally differentiate into plasmablasts and plasma cells resulting in reactivation, lytic replication and production of infectious particles, which infect new naïve B cells completing the viral life cycle and replenishing the EBV reservoir (7, 8). EBV reactivation is tightly controlled by memory T cells, which keep the EBV-load for each individual at a constant level (9–11). During EBV reactivation, epithelial cells of the oropharynx can be reinfected and shed viral particles into the saliva (12), which passes EBV on to new individuals (13).

EBV like human cytomegalovirus encodes a viral IL-10 homologue (ebvIL-10) of cellular IL-10 (cIL-10) (14). ebvIL-10 has 82% amino acid identity to cIL-10 (15, 16), a key anti-inflammatory cytokine of the host with pleiotropic biological activity (17–19). EBV exploits cIL-10 and its viral homologue to evade host immunity and establish a balance with antiviral T cells similar to other viruses that persist in the host (20–23). ebvIL-10 is expressed within a few hours of starting the EBV lytic cycle (24, 25). Approximately 20-30 hours later, in the pre-latent phase, EBV-encoded latent membrane protein-1 (LMP-1) and EBV-encoded small non-coding RNAs (EBERs) upregulate the production of cIL-10 (25–28).

EBV infection seems to be a *conditio sine qua non* for development of multiple sclerosis (MS), the most common chronic inflammatory disease of the central nerve system (CNS) (29–34). The hallmark of MS pathogenesis is an immune attack against axons and their insulating myelin sheath together with disruption of the blood-brain barrier (BBB) (35). As a consequence, inflammation, demyelination, remyelination, neurodegeneration and glial scar formation occur (36). These pathological lesions are either focally or diffusely distributed in the white and grey matter of the brain and spinal cord and result in neurological deficits that differ substantially among patients and over the disease course (37). The global prevalence of MS is rising with nearly 3 million people living with MS worldwide, mostly adults between 20 years and 40 years with females affected twice as likely as males (38, 39). With increasing age, new demyelinated lesions appear less often, but some inflammatory plaques remain. A body of evidence indicates that the immune response is critically involved in MS pathogenesis (40, 41). For development of effective MS therapy and prophylaxis, it is crucial to understand the detailed mechanisms of how EBV drives MS underlying immunopathology.

In this article we discuss how ebvIL-10 and EBV-induced cIL-10 could help the virus evade the immune system and persist in then host while at same time drive inflammatory processes that contribute to MS pathogenesis.

## Biological activity of viral and virus-induced cellular IL-10

Biologically active IL-10 binds with high-affinity to a private receptor subunit (IL-10R1), and with low-affinity to a public receptor subunit (IL-10R2) shared in common with other members of the cytokine class II family (42). IL-10R1 represents the ligand binding subunit of the receptor complex, whereas IL-10R2 is the signaling subunit. Upon binding to IL-10, IL-10R1 induces a conformational change in IL-10R2, permitting IL-10R2 to also bind IL-10. Subsequently, the intracellular Janus tyrosine kinases Jak1 and Tyk2 are activated resulting in phosphorylation of signal transducer and activator of transcription 3 (STAT3), which induces the cellular responses.

The affinity of IL-10R1 for ebvIL-10 is approximately 1000-fold less than for cIL-10 (43). Thus, ebvIL-10 is a selective agonist with impaired binding to the IL-10R1 (16). This implies that cIL-10 and ebvIL-10 share some but not all biological activities. In accordance, ebvIL-10 is unable to signal thymocytes and mast cells, which both express low levels of IL-10R1, whereas cIL-10 signals normally (44–46). Moreover, ebvIL-10 does not upregulate MHC class II molecules on B cells (47). In comparison to cIL-10, ebvIL-10 impacts only weakly on DC function (48). Furthermore, ebvIL-10 can inhibit the effects of cIL-10 on monocytes (49). In fact, ebvIL-10 reduced cIL-10 induced STAT3 phosphorylation to levels similar to monocytes stimulated with ebvIL-10 alone (49). In contrast, ebvIL-10 has retained the ability to increase B cell growth and differentiation (50).

Thus, ebvIL-10 has evolved to retain some but not all functions of cIL-10 and in some circumstances can even compete with cIL-10. Both molecules act in a coordinated fashion, however, to facilitate EBV persistence in the host by balancing viral load and antiviral immune responses.

## Infectious mononucleosis as a prelude to MS

Primary EBV infection in developing countries takes place during childhood and is in general asymptomatic (51). In contrast, primary infection in developed countries occurs during adolescence (11 to 19 years of age) and young adulthood (20 to 24 years of age) and is associated with infectious mononucleosis (IM) (52). IM is a self-limiting disease with fever, sore throat, skin rash, tender lymphoadenopathy and hepatosplenomegaly. These IM symptoms are a consequence of an exaggerated antiviral T cell response involving predominantly CD8+ T cells (53–55). In IM patients, cIL-10 levels as well as the EBV load in circulating B cells are increased compared to healthy controls (56–58). Moreover, EBV-infected B cells dramatically enlarge the CD8+ T cell compartment resulting in up to 50% of CD8+ T cells reacting to lytic EBV antigens (59). The disease severity correlates with both blood EBV load and proliferation of CD8+ T cells (53). Although the blood EBV load is high in both asymptomatic primary infection and IM, only IM patients showed exaggerated T cell responses (60). Strikingly, the risk for MS is absent in EBV-seronegative individuals, increases after EBV infection, is high after IM in adolescence and particularly high after IM in early adulthood (56, 61–66). Accordingly, developed countries experience most cases of IM and have considerably higher prevalences and incidences of MS compared to developing countries (38, 67).

Taken together, primary EBV infection in adolescents and young adults is associated with IM, high blood EBV load, high cIL-10 levels, exaggerated T cell responses, and a high risk for MS.

## Increased EBV load as a risk factor for MS

After primary infection, T cells continuously control and interact with EBV-infected B cells (68). The size of the peripheral EBV reservoir is determined to a large part by the frequency and functional activity of CD8+ T cells eliminating EBV-infected B cells. This loss is countered by periodic virus reactivation and fresh infection of naïve B cells, which are then reprogrammed into latently EBV-infected memory B cells within a few days post infection (3). Healthy EBV-seropositive humans vary greatly in the number of latently infected memory B cells, from 1 to 50 per 10^6^ peripheral B cells (69). However, in each person the levels of latent EBV remains stable over time, defining a steady state or “set point” for each individual (69–71). After IM, the EBV loads in the saliva are persistently high and the pool of latently EBV-infected B cells remains elevated for a considerable time period as compared to healthy EBV carriers without a record of IM (53, 72).

An elevated set point of the EBV load may facilitate and drive MS. In accordance, peripheral blood mononuclear cells from MS patients harbor increased numbers of latently EBV-infected B cells compared to healthy controls (73). Moreover, monoclonal antibodies directed against CD20+ cells, which reduce MS pathology, spare plasma cells and do not reduce the amount of immunoglobulin but remove B cells including those with latent EBV thereby decreasing the set point (74, 75). Accordingly, the effectiveness of anti-CD20 treatment in MS patients is probably not based on the reduction of autoantibody levels but rather on depleting B cells that drive MS by presenting auto-antigens (76). Consistent with this view, individuals overexpressing B-cell activating factor (BAFF), which increases B-cell activation, differentiation, and survival, have a higher risk for MS (77, 78). Moreover, adoptive transfer of EBV-specific T cells, a promising strategy in MS treatment, reduces the pool size of latently EBV-infected B cells (79–81). The observation that high serum titres of antibodies against EBV nuclear antigen 1 (EBNA1), an essential viral protein for EBV latency, enhance the risk for MS supports this notion (82). Importantly, anti-EBNA1 antibody titres were already significantly elevated 5 or more years prior to MS onset suggesting that a high EBV set point leads MS and not vice versa (83, 84). In MS, the anti-EBNA1 IgG titre correlate inversely with the frequency of EBV-specific CD8^+^ T cells supporting the notion that these immune cells control EBV reactivation and the set point of the virus load (85). Intriguingly, the anti-EBNA1 antibody levels after primary EBV infection are to a large part genetically determined, for example by variants of MHC class II molecules (86–89). Moreover, co-infection with Malaria increases the circulating EBV load (90). Thus, genetic and environmental factors such as coinfections with other pathogns or gut microbiota influence the EBV load and MS development, for example through modulating the cytokine network and triggering EBV reactivation (55, 68, 91–96).

Altogether, these data support the idea that an increased load of latently EBV-infected memory B cells after primary infection is due to genetic and environmental factors and represents an important risk factor for MS.

## IL-10 dependent mechanisms adjusting the set point of the EBV load

**Figure 1:**
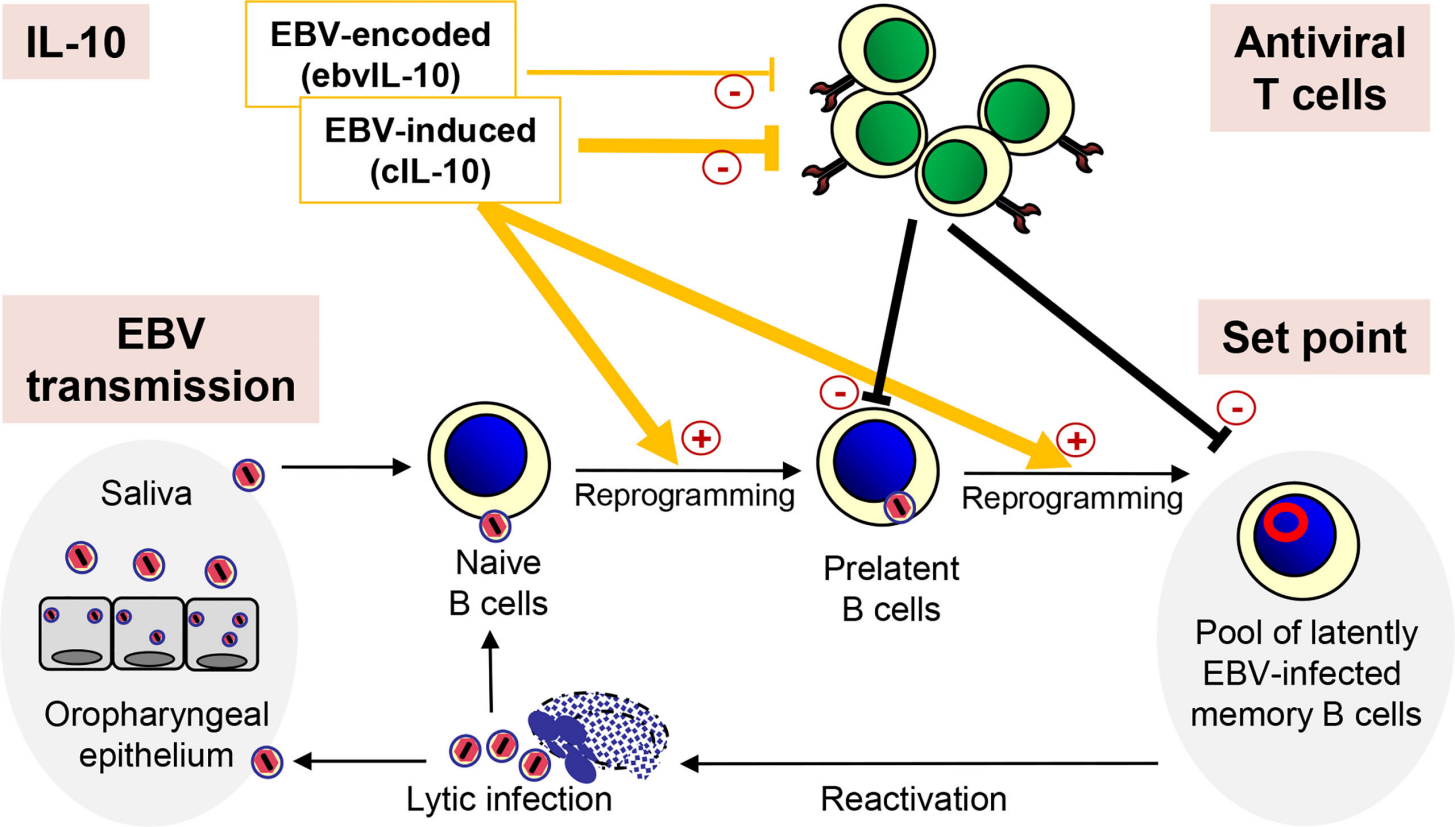
Regulation of the EBV load by IL-10. After transmission, EBV infects naïve B cells, which are then reprogrammed and replenish the pool of latently EBV-infected memory B cells. Occasionally, EBV reactivates and lytically infected plasma cells produce new virus particles, which infect new naïve B cells completing the viral life cycle. After reinfection, epithelial cells of the oropharynx shed viral particles into the saliva, which passes EBV on to new individuals. Importantly, the coordinated action of EBV-induced cIL-10 and ebvIL-10 regulates the individual set point of the EBV load not only by reprogramming EBV-infected naïve B cells but also by regulating the activity of antiviral T cells that eliminate newly infected B cells.

The coordinated action of EBV-induced cIL-10 and ebvIL-10 regulates the set point of the EBV load not only by enhancing differentiation, proliferation and survival of EBV-infected naive B cells but also by regulating the activity of antiviral T cells (**Figure 1**). Dysregulation of this delicate balance could facilitate the spread of latent EBV in the memory B cell compartment thereby increasing the set point and the risk for MS. In line with this view, MS patients show decreased T cell reactivity against autologous lymphoblastoid cell lines (LCLs) indicating an attenuation of the immune surveillance (97). LCLs are EBV-transformed B-cell lines that continuously proliferate *in vitro*. They can be easily established by EBV infection or derived spontaneously *ex vivo* from peripheral blood B lymphocytes in the absence or inhibition of T and NK cells. Emphasizing the regulatory role of IL-10 in this context, in a mouse model gammaherpesvirus-induced IL-10 was required for expansion and differentiation of latently infected B cells, while at the same time interfering with the activity of antiviral CD8+ T cells (98).

It has been shown that cIL-10 can inhibit CD8+ T cell function directly thereby facilitating persistent virus infection (99). EBV-induced cIL-10 abrogates the capacity of T cells to inhibit the outgrowth of autologous LCLs (100). Similarly, ebvIL-10 has been reported to interfere with elimination of newly infected B cells by CD8^+^ T cells (100–102). As a potential mechanism, both EBV-induced cIL-10 and ebvIL-10 downregulate the transport of peptides into the endoplasmic reticulum resulting in reduced surface expression of MHC class I molecules and presentation of viral epitopes to CD8+ T cells (103). However, other investigators reported that ebvIL-10 can also enhance the activity of EBV-specific CD8+ T cells (104). Moreover, cIL-10-stimulatory effects on functionality of CD8+ T cells have also been described (105–107). Thus, the coordinated action of ebvIL-10 and EBV-induced cIL-10 may allow minimal activity of EBV-specific CD8+ T cells that is required to push EBV back into latency and prevent outgrowth of B cell lymphomas due to opportunistic expansion of latently infected B cells.

In conclusion, ebvIL-10 and EBV-induced cIL-10, which are regulated by genetic host factors and environmental cues, play a crucial role in defining the set point of the individual EBV load in the periphery.

## Pathological consequences of a disproportionally increased EBV load

Individuals with high EBV load display virus-specific CD8+ T cells showing signs of exhaustion such as surface upregulation of programmed cell death protein 1 (PD-1) (108). In a vicious cycle, EBV-specific CD8+ T cells confronted with a high EBV load may be further stimulated to proliferate without being able to re-establish a balance (68, 109). As a consequence, control of EBV reactivation in MS patients becomes defective resulting in further expansion of latently EBV-infected B cells that may also include autoreactive B cells contributing to MS pathogenesis (85). Possibly due to T cell exhaustion the impairment of EBV control by antiviral T cells in MS patients worsens with age (110) whereas strong CD8+ EBV-specific T cell responses are found in patients with early and active MS (111, 112).

A high EBV load is associated with frequent but stochastic reactivation that is randomly distributed in lymphoid tissue of the host. The local release of infectious EBV particles could create an inflammatory microenvironment, periodically flaring up before subsiding again. These oscillating stimulatory events increase the likelihood of breaking self-tolerance and molecular mimicry (113). An increased number of B cells could present EBV-derived peptides that contain molecular mimicry motifs allowing stimulation of auto-reactive T cells (114). In accordance, peripheral memory B cells drive proliferation of CD4+ T cells that recognize peptides expressed in MS brain lesions (115). Indeed, homologies between EBV-encoded proteins on the one hand and myelin and other CNS antigens on the other have been found (34). T cell clones from MS patients recognizing myelin basic protein are activated by peptides derived from EBV-encoded DNA polymerase, which is expressed during lytic infection (116, 117). EBNA-1 is not only expressed in all forms of latent infection but also during the lytic phase of infection (118). A recent study demonstrated that antibodies against EBNA-1 cross-react with glial cell adhesion protein (GlialCAM), a self-antigen expressed in the CNS (119, 120). EBV-specific B cells cross the BBB and form ectopic lymphoid-like structures, which are often found in infection and autoimmunity (121). After undergoing somatic hypermutation, B cells that produce cross-reactive antibodies with high affinity for GlialCAM are selected by ENBA-1 specific CD4+ T follicular helper cells and follicular dendritic cells. These cross-reactive antibodies subsequently damage myelin-producing glial cells (119). Moreover, clonally expanded EBNA1-specific CD4+ T cells cross-reacting with myelin are observed in MS patients (122).

Taken together, a high EBV load in the periphery could facilitate pathogenic B–T cell interactions resulting in stimulation and accumulation of auto-reactive immune cells that cross the BBB and drive demyelination.

## Pathological role of IL-10 in CNS autoimmunity

B cells crossing the BBB represent an important immune axis between periphery and CNS of MS patients (123–125). Thus, the latently EBV-infected B cell reservoir in the CNS is continuously replenished by the immune axis between periphery and CNS. The presence of EBV-infected B cells in the CNS of MS patients has been reported by numerous studies (126). In ectopic lymphoid-like structures of the CNS, latently EBV-infected B cells differentiate into plasma cells, resulting in EBV reactivation, lytic infection and stimulation of cytotoxic CD8+ T cells (127–129). EBV gene expression patterns found in the brain of MS patients support the idea of EBV reactivation and EBV entry into the lytic cycle (130). Moreover, cytotoxic CD8+ T cells interacting with plasma cells lytically infected with EBV were observed in inflammatory white matter lesions and meninges from post-mortem MS brain samples (111). In these lesions, CD8+ T cells recognizing lytic EBV antigens tended to be more frequent than those recognizing EBV latent proteins (131). Thus, ebvIL-10 and EBV-induced cIL-10 are likely released in the CNS thereby maintaining a reservoir of latently EBV-infected B cells that stimulate pathogenic T cells also at this site. In accordance, cIL-10 drives CNS inflammation by promoting survival of pathogenic T cells in mouse models of CNS autoimmunity (132, 133). Accordingly, periodic reactivation of EBV and the release of infectious virus particles could create an inflammatory environment that facilitates MS immunopathology. In line with this view, lytic EBV was restricted to chronic MS plaques (134).

## Concluding remarks

**Figure 2:**
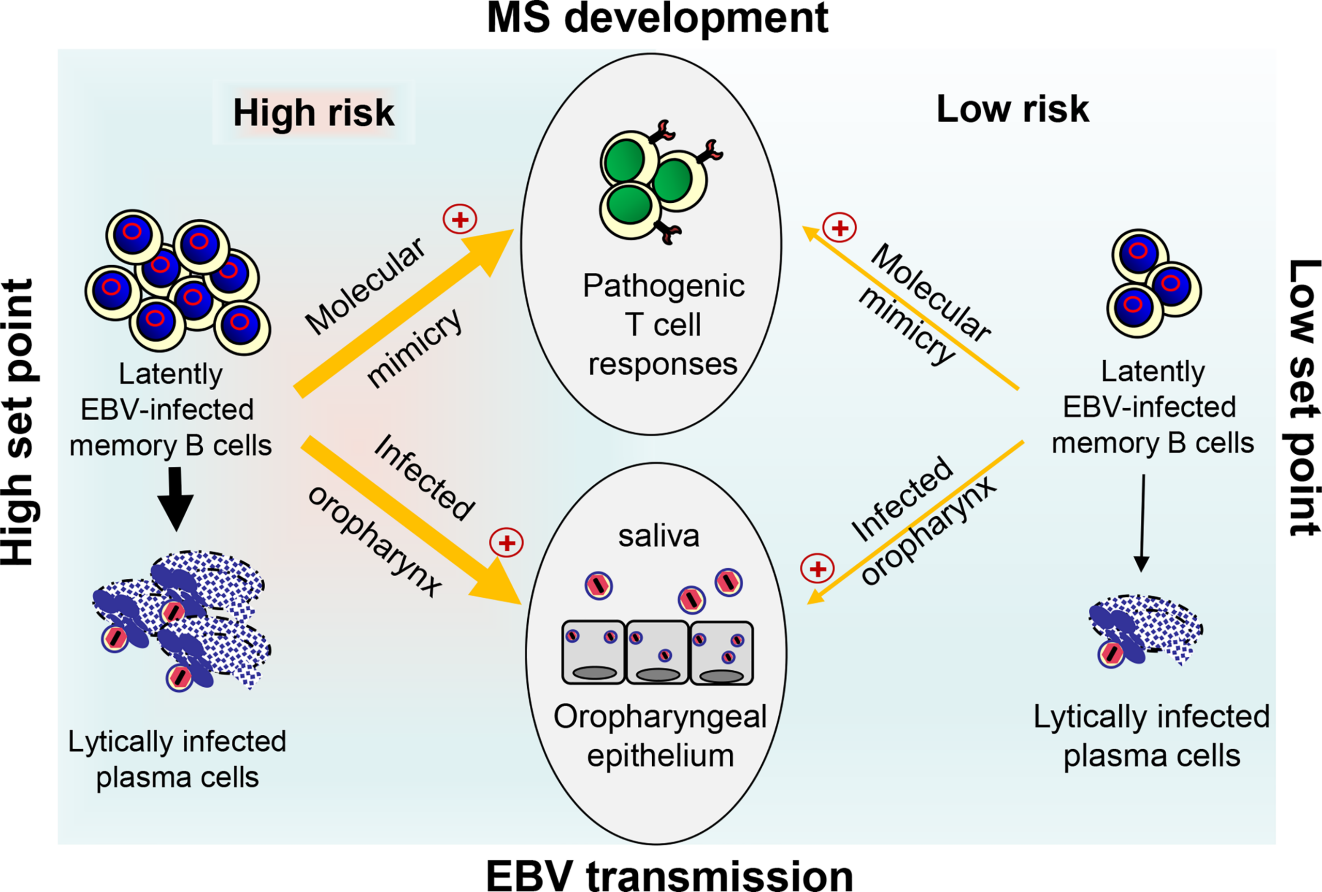
Link between EBV load, risk for MS development, and EBV transmission. At a high set point of the EBV load (left side), reactivation of EBV in latently infected memory B cells and release of infectious EBV particles occurs frequently in lymphoid tissue. This may allow not only persistent oral shedding with high EBV transmission but also facilitate pathogenic B–T cell interactions thereby drastically increasing the risk for pathogenic T ell responses that initiate and drive MS through molecular mimicry. The latter occurs when B cells present EBV-derived peptides that are similar to self-peptides found in CNS antigens. At a low set point (right side), however, EBV reactivates only rarely resulting in inefficient EBV transmission due to reduced oral shedding and only a low risk for MS.

The coordinated action of both EBV-induced cIL-10 and ebvIL-10 plays an important role in viral immune evasion and virus persistence. By reprogramming EBV-infected naïve B cells and regulating the antiviral T cell responses, these cytokines could define the set point of the latently EBV-infected B cell reservoir, which varies from person to person but remains stable in each person over time. At a low set point, EBV reactivates only rarely and is not efficiently passed on from one person to another due to minimal oral shedding coincident with low MS risk (**Figure 2**). In striking contrast, if the set point is too high as observed after IM, reactivation and release of infectious EBV particles occurs frequently in lymphoid tissue that harbors latently EBV-infected memory B cells creating an inflammatory microenvironment. This may allow not only persistent oral shedding with high virus transmission but also drastically increases the risk for pathogenic immune responses that initiate and drive MS (**Figure 2**). In the future, it might be sensible to calculate the risk of young adults with a previous record of IM by quantifying the EBV load and develop prophylactic and therapeutic measures that adjust the set point of a disproportionally high EBV load downwards. Most importantly, an effective vaccine against EBV infection could prevent MS and its deleterious consequences.

## Data availability statement

The original contributions presented in the study are included in the article/supplementary material, further inquiries can be directed to the corresponding author.

## Author contributions

GS, MOA, and MR drafted and corrected the text and figure. All authors contributed to the article and approved the submitted version.

## Funding

This work was supported by the Berlin Institute of Health (BIH).

## Acknowledgments

The authors apologize that many research articles with relevance to the field were not included in this review due to space limitations.

## Conflict of interest

The authors declare that the research was conducted in the absence of any commercial or financial relationships that could be construed as a potential conflict of interest.

## Publisher’s note

All claims expressed in this article are solely those of the authors and do not necessarily represent those of their affiliated organizations, or those of the publisher, the editors and the reviewers. Any product that may be evaluated in this article, or claim that may be made by its manufacturer, is not guaranteed or endorsed by the publisher.
